# Colonic Lipoma Mimicking Gastrointestinal Stromal Tumor: A Case Report of a Diagnosis Pitfall

**DOI:** 10.7759/cureus.92566

**Published:** 2025-09-17

**Authors:** Nicolas Ascencio Jaramillo, Sergio Darley Leon Castro, Jose Omar Zorrilla Lara

**Affiliations:** 1 Emergency Department, Hospital Universitario Fundación Valle del Lili, Cali, COL; 2 General Surgery, Hospital Universitario del Valle “Evaristo García,” Universidad del Valle, Cali, COL; 3 Oncologic Surgery, Hospital Universitario del Valle “Evaristo García,” Universidad del Valle, Cali, COL

**Keywords:** colonic lipoma, gastrointestinal stromal tumors (gists), intestinal obstruction, minimally invasive laparoscopy, persistent abdominal pain

## Abstract

Colonic mesenchymal tumors represent a diagnostic challenge due to their rarity and the overlap of clinical and imaging features with other subepithelial lesions, particularly gastrointestinal stromal tumors (GISTs). Histopathological evaluation remains essential to guide appropriate management and establish prognosis.

We report the case of a 64-year-old woman with a three-year history of abdominal distension, altered bowel habits, and occult gastrointestinal bleeding. Repeated colonoscopies revealed a large subepithelial lesion in the transverse colon, highly suggestive of GIST, though repeated biopsies were nondiagnostic. As symptoms persisted and intestinal obstruction developed, the patient underwent a laparoscopic left hemicolectomy with intracorporeal anastomosis. Final histopathological analysis revealed a benign colonic lipoma. The patient’s postoperative course was uneventful, with marked improvement in quality of life.

This case illustrates the clinical difficulty of distinguishing between GISTs and colonic lipomas, as endoscopic imaging and biopsy often fail to yield a definitive diagnosis. Tumor size, endoscopic features, and obstructive symptoms guided the therapeutic decision. Surgical resection resolved the obstruction and provided a definitive diagnosis, offering both clinical and psychological benefits.

This case highlights the importance of considering colonic lipoma in the differential diagnosis of large subepithelial colonic lesions and reinforces the role of surgery in both treatment and diagnostic certainty.

## Introduction

Gastrointestinal stromal tumors (GISTs) represent a rare entity, with an estimated incidence of 10-20 cases per million people per year. They predominantly affect adults between 50 and 70 years of age, although exceptional cases have been reported in younger individuals [1].

Clinically, GISTs often present with nonspecific symptoms such as anemia, weight loss, abdominal pain, or gastrointestinal bleeding, which may delay diagnosis and contribute to clinical uncertainty [2]. Gastric GISTs tend to remain asymptomatic until they reach a considerable size or are incidentally discovered during imaging performed for unrelated reasons, highlighting the diagnostic uncertainty that often necessitates histopathological confirmation [3].

Cross-sectional imaging, including computed tomography (CT) and magnetic resonance imaging (MRI), is the primary modality used to evaluate tumor extent and characteristics. However, histopathological analysis remains the only method capable of establishing a definitive diagnosis [3,4].

Because of their radio-resistant nature and poor response to conventional chemotherapy, the treatment of choice for localized GISTs is complete surgical resection, particularly in the absence of metastatic disease [5].

In contrast, colonic lipomas are benign mesenchymal neoplasms and represent the second most common non-epithelial tumor of the colon after adenomas. Histologically, they are composed of mature adipose tissue located within the intestinal wall, most frequently in the submucosa [6]. Their reported prevalence in clinical and autopsy studies ranges from 0.2% to 4.4%, reflecting their relatively uncommon but not exceptional occurrence [7].

Although most colonic lipomas remain asymptomatic, approximately one-quarter of patients develop clinical manifestations, particularly when the tumor exceeds 2 cm in diameter. Symptoms may include abdominal pain, altered bowel habits, and mild rectal bleeding. In rare cases, complications such as intussusception or intestinal obstruction may arise, requiring urgent surgical intervention [8].

## Case presentation

A 64-year-old female with a history of arterial hypertension managed with calcium channel blockers presented with a three-year history of intermittent abdominal distension, altered bowel habits, and occult blood in stool confirmed on coprological testing.

A colonoscopy in 2021 revealed a smooth-edged mass measuring approximately 40 mm in the left transverse colon, occupying more than 80% of the intestinal lumen. The remaining colonic mucosa appeared normal, with preserved caliber and vascular pattern. Biopsies obtained at that time were inconclusive.

A repeat colonoscopy in 2022 described a 4-5 cm wide-based, lobulated, subepithelial lesion with an ulcerated surface. Endoscopic marking with India ink was performed for surgical localization. Histopathology demonstrated extensive fibrinoid necrosis, vascular proliferation, interstitial fibrosis, and mixed inflammatory infiltrates predominantly composed of neutrophils, resulting in a non-specific diagnosis of ulceration.

Given the persistence of symptoms and the absence of histological confirmation, the patient was referred to oncologic surgery in 2023. The lesion was considered highly suggestive of a GIST. Further evaluation was recommended, including contrast-enhanced abdominal CT, which demonstrated the presence of the colonic mass, chest X-ray that showed no evidence of metastatic involvement, and a preoperative assessment. The case was subsequently discussed at a multidisciplinary tumor board.

In January 2024, the patient presented to the emergency department with melena-like stools, nausea, vomiting, and oral intolerance, consistent with intestinal obstruction. Given the obstructive symptoms (oral intolerance with emesis), along with hydroaeric levels on abdominal CT and findings on physical examination of a distended, tender abdomen, urgent surgical intervention was indicated due to the risk of major complications.

**Figure 1:**
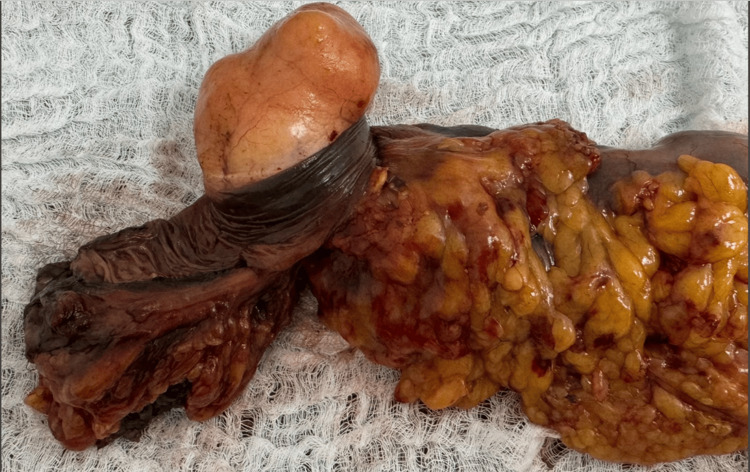
Hemicolectomy of the transverse colon with a tumor.

**Figure 2:**
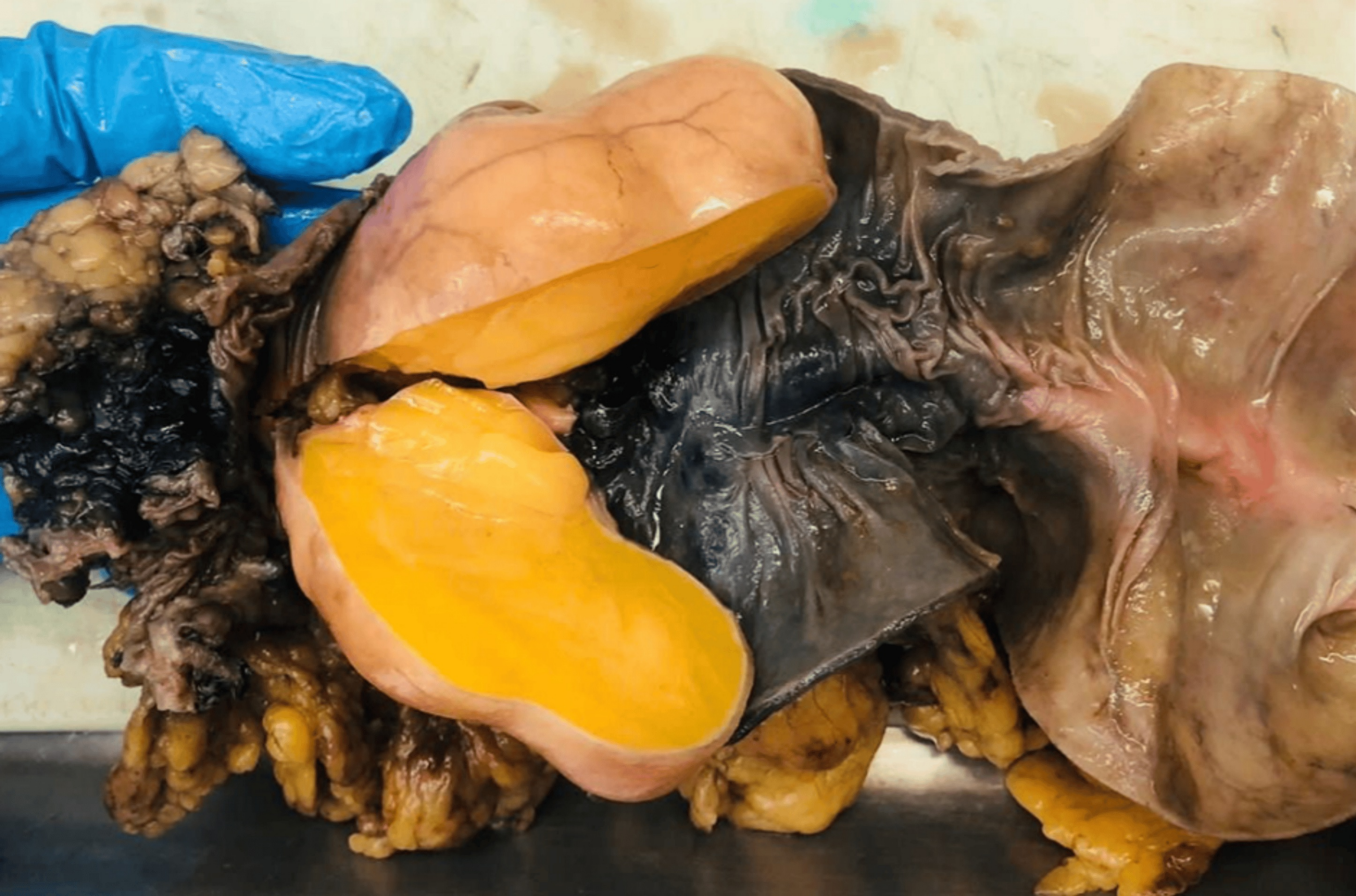
Transverse colon tumor (lipoma) approximately 8 cm in diameter.

**Figure 3:**
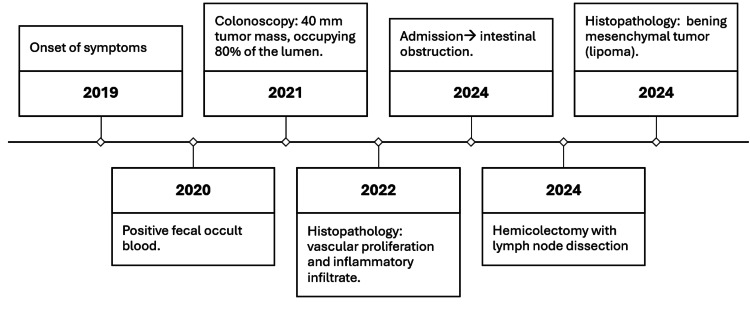
Case presentation timeline.

After obtaining informed consent, a laparoscopic left hemicolectomy with lymph node dissection and intracorporeal colo-colonic anastomosis was performed. Using a 60-mm Signia stapler, the lesion was resected with adequate proximal and distal oncological margins, followed by lymphadenectomy and construction of an intracorporeal colo-colonic anastomosis. Intraoperatively, an 8-cm obstructive tumor was identified in the transverse colon near the splenic flexure, associated with proximal colonic dilation (Figure 1, Figure 2). No hepatic metastases, ascites, or peritoneal implants were observed. The postoperative course was uneventful (Figure 3).

Histopathological analysis revealed a benign mesenchymal tumor composed of mature adipocytes without atypia, consistent with colonic lipoma. 

The patient recovered progressively and was discharged with outpatient follow-up by general surgery and oncology. Resolution of diagnostic uncertainty after multiple invasive procedures provided significant clinical improvement and emotional relief for the patient and her family.

## Discussion

Mesenchymal tumors of the colon pose a significant diagnostic challenge due to their low frequency and the overlap of clinical and imaging findings with other subepithelial lesions, particularly GIST. In the present case, the tumor’s size and endoscopic appearance strongly suggested a GIST, influencing both diagnostic evaluation and therapeutic planning. However, definitive histopathological analysis confirmed a benign lesion consistent with colonic lipoma [9,10].

GISTs are characterized by variable biological behavior and an unpredictable risk of malignancy. Their diagnosis requires histopathological confirmation supported by immunohistochemistry, with c-KIT (CD117) being the essential marker. Complete surgical resection with negative margins remains the cornerstone of treatment, given their poor response to conventional chemotherapy and radiotherapy [11].

In contrast, colonic lipomas are benign lesions that, although usually asymptomatic, may become clinically significant when large, as in this patient. Previous reports of giant colonic lipomas have described their potential to mimic malignant tumors; however, confusion with colonic GIST is exceptionally rare, which underscores the diagnostic relevance of this case [12-14].

Prolonged diagnostic uncertainty due to inconclusive biopsies significantly affected the patient's quality of life. This aspect underscores the human dimension of surgical intervention: beyond technical resolution, surgery provided diagnostic certainty and substantial emotional relief to both the patient and her family [15].

The ideal diagnostic approach for colonic subepithelial lesions should integrate colonoscopy with targeted biopsies when technically feasible, complemented by cross-sectional imaging such as contrast-enhanced CT or MRI to further characterize the lesion and its relationship with surrounding structures. This case highlights the diagnostic complexity of colonic mesenchymal tumors and reaffirms the role of surgery as a definitive management strategy. Beyond its therapeutic value, surgical resection contributed to restoring the patient’s overall well-being. The confirmation of colonic lipoma, in a context initially suggestive of GIST, not only represents a favorable clinical outcome but also enriches the literature by emphasizing the importance of a comprehensive and multidisciplinary approach to such cases [12,13].

## Conclusions

Colonic subepithelial lesions may closely mimic malignant tumors such as GIST, making histopathological confirmation essential. This case underscores the limitations of repeated endoscopic biopsies in large or ulcerated lesions, where diagnostic uncertainty may persist. Surgical resection is both therapeutic and diagnostic, particularly in symptomatic or complicated cases, ensuring oncological safety while preventing life-threatening complications such as obstruction or hemorrhage.
